# Reaction time and onset of psychological distress: the UK Health and Lifestyle Survey

**DOI:** 10.1136/jech-2015-206479

**Published:** 2016-02-04

**Authors:** Catharine R Gale, Alicia Harris, Ian J Deary

**Affiliations:** 1 Department of Psychology, Centre for Cognitive Ageing and Cognitive Epidemiology, University of Edinburgh, Edinburgh, UK; 2 MRC Lifecourse Epidemiology Unit, University of Southampton, Southampton, UK; 3 Department of Psychology, University of Edinburgh, Edinburgh, UK

**Keywords:** COGNITION, Cohort studies, DEPRESSION, MENTAL HEALTH

## Abstract

**Background:**

Cross-sectional studies have shown that depression is often accompanied by less efficient cognitive function, as indicated by slower speed of information processing. The direction of effect is unclear. We investigated prospectively whether slower processing speed, as indexed by longer simple or choice reaction time, is associated with an increased risk of psychological distress.

**Methods:**

Participants were 3088 men and women aged 18 and over who had taken part in the UK Health and Lifestyle Survey. Simple and choice reaction time was measured in the baseline survey. Symptoms of psychological distress were assessed at baseline and at the 7-year follow-up survey with the 30-item General Health Questionnaire (GHQ).

**Results:**

In unadjusted models, a SD slower simple or choice reaction time at baseline was associated with ORs for psychological distress (≥5 on GHQ) at follow-up of 1.14 (1.06 to 1.23; p<0.001) or 1.13 (1.04 to 1.22; p=0.002), respectively. Further adjustment for age, sex, social class, educational attainment, health behaviours, number of chronic physical illnesses present, neuroticism and GHQ score at baseline had only slight attenuating effects on these associations. In fully adjusted models, a SD slower simple or choice reaction time at baseline was associated with ORs for psychological distress of 1.11 (1.02 to 1.21; p=0.017) or 1.11 (1.00 to 1.24; p=0.048), respectively.

**Conclusions:**

Slower processing speed may be a risk factor for the development of psychological distress. Future studies should explore the extent to which slower processing speed explains previously demonstrated associations between lower intelligence and poorer mental health.

## Introduction

Several cross-sectional studies have shown that depression is often accompanied by less efficient cognitive function, as indicated by slower speed of information processing. People diagnosed with major depression and those who report symptoms of depression have been shown to perform less well on a range of measures of processing speed, such as the Processing Speed Index of the Wechsler Adult Intelligence Scale-III,^1^ inspection time^2^ and reaction time.^3^ Slowed processing speed has also been found in a small cross-sectional study of depressed children.^4^

It is unclear from these studies whether the slower processing speed observed in people with depression was a consequence of the disorder or whether it preceded the onset of illness and was in fact a risk factor for it. Findings that people who have recovered from an episode of depression tend to have slower processing speed than healthy controls suggest the deficit might be trait-dependent rather than state-dependent,^5^ ^6^ but evidence from longitudinal studies is needed to establish whether slower processing speed is a risk factor for onset of psychological distress. To our knowledge, there has been only one such study to date. In a study of 705 adolescents, those who had slower choice reaction time at age 16 years had more symptoms of anxiety and depression, as measured by scores on the General Health Questionnaire (GHQ), at age 36, independently of baseline GHQ score and other potential confounding factors.^7^ This observation is consistent with findings that lower scores on tests of intelligence in youth increase the risk of later diagnosis with depression or anxiety and of reporting symptoms of these disorders.^8–13^ Reaction time and scores on other measures of processing speed are moderately highly correlated with intelligence such that people with higher intelligence tend to process information faster,^14^ so it is plausible that processing speed might be a risk factor for the onset of psychological distress. In contrast to tests of intelligence, tests of reaction time are almost knowledge-free and performance on them is less likely to be influenced by education or socioeconomic status, and less prone to practice effects. It may therefore provide a purer indicator of the brain's processing efficiency than scores on an intelligence test.

We used data from the UK Health and Lifestyle Survey (HALS) to investigate the longitudinal relationship between processing speed, as measured by reaction time, and later onset of psychological distress, as measured by scores on the GHQ, in men and women aged 18 and over, controlling for baseline GHQ score and a range of other potential confounding factors.

## Methods

### Participants

The target population for the UK HALS was the adult population of England, Wales and Scotland aged 18 or older in 1984–1985.^15^ In total, 12 254 addresses were randomly selected from UK electoral rolls and 1 individual between the ages of 18 and 99 years was chosen from each household. In total, 9003 participants were interviewed at home and 7414 consented to a further visit from a nurse. Seven years later, in 1991–1992, a follow-up survey was carried out during which 5352 of the 9003 original participants were interviewed at home. Ethical approval for the HALS surveys was received from the British Medical Association Ethical Committee.

### Measures

#### Reaction time

Simple and choice reaction time was assessed at the initial survey by a nurse using a portable battery-operated testing device.^14^ ^16^ Simple reaction time was measured as the time taken for a participant to press a button as quickly as possible in response to a stimulus. Participants rested their finger of choice on the button marked ‘0’ on the battery-operated device and pressed it as quickly as possible after the stimulus of ‘0’ appeared on the LCD screen of the device. There were 8 practice trials followed by 20 test trials where the mean and SD of the 20 reaction times were recorded in milliseconds for each participant. The interstimulus interval varied between 1 and 3 s.

Choice reaction time was measured as the time taken to press one of four buttons corresponding to one of four numbers. Participants were required to rest their second and third fingers on both of their hands on keys numbered 1, 2, 3 and 4. A number between one and four was presented on the LCD screen of the device and participants were required to press the corresponding button as quickly as possible. There were 8 practice trials and 40 test trials where each digit was randomly presented 10 times at interstimulus intervals of between 1 and 3 s. Data on the mean and SD of the correct test trials only were recorded. In total, ≤1% of participants who were present at the reaction time measuring session did not provide data for either simple or choice reaction time.

#### Psychological distress

Psychological distress at the initial survey and at follow-up was assessed using the 30-item GHQ.^17^ The GHQ was completed by the participants at home after the visit by the nurse and posted back to the researchers. Each of the 30 questions in the GHQ was answered using a four-point Likert scale noting the degree to which the respondent has experienced a particular symptom. There were four response options: ‘not at all’, ‘no more than usual’, ‘rather more than usual’, ‘much more than usual’ (0–3). Scoring was based on the 0–0/1–1 method so that ‘not at all’ and ‘no more than usual’ were coded as 0 (the symptom is not experienced) and ‘rather more than usual’ and ‘much more than usual’ are scored as 1 (the symptom is experienced). This results in a score ranging from 0 to 30, with higher scores indicating greater distress. The presence of psychological distress at follow-up was defined as a score on the 30-item GHQ of 5 or more. The Cronbach α for the GHQ was 0.91 at baseline and 0.89 at follow-up indicating good internal consistency.

#### Covariates

We chose age, occupational social class, educational attainment, neuroticism, health behaviours (smoking, alcohol consumption, frequency of fruit and vegetable consumption, and physical activity), and the presence of chronic physical disease as covariates that might potentially confound any association between reaction time and psychological distress. Data on all covariates were taken from the initial survey. The UK Registrar General's classification was used to derive social class in six categories based on current or most recent occupation of the participant (or in the case of married women, her husband).^18^ Educational attainment was defined as the highest qualification obtained and classified in five categories, ranging from no qualifications to degree. Neuroticism was assessed using the Eysenck Personality Inventory (EPI).^19^ Items were rated as yes (score=1) or no (score=0) creating a total score of 24 with higher scores representing higher levels of neuroticism. The Cronbach α for neuroticism was 0.84 indicating good internal consistency. Smoking status was categorised as never-smoker, ex-smoker, current smoker. Participants provided information on frequency of eating fruit or vegetables in six categories (never, less than once a week, once or twice a week, most days, once a day, more than once a day). Participants provided information on alcohol consumption in the last week from which units of alcohol was derived. Participants were presented with a list of 17 different physical activities—such as keep fit, jogging, tennis, swimming, football, cycling—and asked how long they had spent on each in the past 2 weeks. An overall measure of physical activity was calculated by adding the time spent on each activity and categorising it into four groups: no activity/up to 30 min per week/up to 2 h per week/over 2 h per week. Participants were asked about the presence of any long-standing physical illness, disability or infirmity. We summed the number of physical illnesses reported as a measure of burden of chronic physical disease.

### Analytical sample

Of the 9003 people who took part in the initial survey, 5352 (59%) were interviewed in the follow-up survey of whom 4483 were visited by a nurse and given the GHQ to complete and post back after the visit. In total, 3626 completed and returned the GHQ at follow-up. Our analyses are based on 3088 participants who had complete data on GHQ score at follow-up and reaction time and all the covariates at baseline. To check for the presence of bias due to the exclusion of those with missing covariate data, we also carried out an analysis based on the 3626 people with outcome data, in which multivariate multiple imputation was used to impute missing covariate data.

### Statistical analysis

We used analysis of variance and the χ^2^ test to examine the participants’ baseline characteristics according to psychological distress at follow-up. Spearman correlation coefficients were used to examine the correlations between these characteristics. We used logistic regression to examine the relationship between simple and choice reaction time at baseline (expressed as a SD increase) and psychological distress at follow-up (GHQ score ≥5), with adjustment for the potentially confounding factors total GHQ score at baseline, age, alcohol consumption, smoking status, physical activity, neuroticism, educational attainment, social class and number of chronic physical diseases. In the logistic regression analyses, smoking status, educational attainment and social class were treated as categorical variables. Preliminary analysis indicated that the relationships between simple and choice reaction time at baseline and psychological distress at follow-up did not differ by sex (p for interaction terms >0.3), so we analysed both sexes together and adjusted for sex. To investigate possible bias due to missing data, we used multiple multivariate imputation to impute values in any covariates with missing values.^20^ Imputation models included reaction time, GHQ score at follow-up and the potential confounding variables. We used STATA software to generate 10 imputation data sets. The multiple multivariate imputation approach creates a number of copies of the data (in this case 10 copies) each of which has missing values imputed based on available data and with an appropriate level of randomness using chained equations. The final estimates are obtained by averaging across the estimates from each of these 10 data sets using Rubin's rules and taking into account the uncertainty in the imputation as well as uncertainty due to random variation.

## Results

Table 1 shows the characteristics of the participants at baseline according to the presence or absence of psychological distress at follow-up 7 years later. Psychological distress at follow-up was more common in women, and in those who at baseline had slower simple or choice reaction time, higher scores for neuroticism, higher scores on the GHQ, more chronic physical illnesses and were ex-smokers or current smokers.

**Table 1:** Baseline characteristics of the study participants according to presence of psychological distress at follow-up (n=3088)

	Psychological distress (GHQ-30 ≥5)		p Value
Characteristics	No (n=2219)	Yes (n=869)	
Age (years), mean (SD)	45.3 (15.2)	44.7 (15.6)	0.30
Female, n (%)	1250 (56.3)	537 (61.8)	0.006
Simple reaction time, mean (SD)	321.3 (102.7)	336.9 (121.6)	0.003
Choice reaction time, mean (SD)	642.6 (109.2)	656.2 (114.0)	0.002
Manual social class, n (%)	1223 (55.1)	491 (56.1)	0.49
No qualifications, n (%)	1046 (47.1)	436 (50.2)	0.13
GHQ-30 score, median (IQR)	1 (0–3)	4 (1–9)	<0.001
Neuroticism, mean (SD)	7.68 (4.64)	11.6 (4.82)	<0.001
Ever smoked, n (%)	1321 (59.5)	556 (64.0)	0.023
Units of alcohol last week, median (IQR)	3 (0–10)	3 (0–10)	0.39
Fruit or vegetables eaten once or twice a week or less often, n (%)	433 (19.5)	193 (22.2)	0.09
No time spent on physical activity in last fortnight, n (%)	1318 (59.4)	513 (59.0)	0.69
Two or more chronic physical illnesses, n (%)	616 (27.8)	325 (37.4)	<0.001

GHQ, General Health Questionnaire.

In general, people with a slower mean simple reaction time tended to have a slower mean choice reaction time (r=0.59, p<0.001).

Rank order correlations between mean simple or choice reaction time and the covariates at baseline and total score on the GHQ-30 at follow-up are shown in the online supplementary table. Slower simple or choice reaction time was associated with older age, lower educational attainment, lower social class, higher neuroticism, lower alcohol intake, being less physically activity, eating fruit or vegetables less frequently and having more chronic physical diseases, and higher scores on the GHQ-30 both at baseline and follow-up. Choice reaction time, but not simple reaction time, was slower in ex-smokers or current smokers.

Compared with the 3088 people with complete data on all variables of interest who made up our analytical sample, the 5915 people who were excluded due to loss to follow-up or missing data were older (mean age 46.2 vs 45.1 years, p=0.01), had slower mean choice and simple reaction time (679.8 vs 646.4 and 351.9 vs 325.7, respectively, both p<0.001), and a higher proportion came from a manual social class background (59.3% vs 55.1%, p=0.001), had no educational qualifications (54.5% vs 48%, p<0.001), or scored ≥5 on the GHQ at baseline. To check for the presence of bias in our estimates due to the exclusion of those with missing covariate data, we carried out an analysis based on the 3626 people with outcome data, in which multiple imputation was used to impute missing covariate data.

Table 2 shows ORs for psychological distress at follow-up for a SD slower (ie, longer) simple or choice reaction time at baseline, first unadjusted and then adjusted for covariates, first in the sample with complete data and then in the sample for whom we imputed missing covariate data. Looking first at the sample with complete data, in unadjusted models, a SD slower simple or choice reaction time at baseline was associated with ORs for psychological distress of 1.14 (1.06 to 1.23; p<0.001) and 1.13 (1.04 to 1.22; p=0.002), respectively. Adjustment for age, sex and total GHQ score at baseline had a small attenuating effect on the association between simple reaction time and psychological distress but slightly strengthened the association between choice reaction time and psychological distress. Further separate adjustment in successive models for socioeconomic factors (social class and education), health behaviours (smoking, alcohol intake and physical activity, frequency of fruit or vegetable consumption), neuroticism and chronic physical illnesses at baseline had only small attenuating effects. In final models adjusting for all covariates, the association between slower reaction time at baseline and increased likelihood of psychological distress at follow-up persisted, with little attenuation from the unadjusted models. For a SD slower simple reaction time at baseline, the multivariate-adjusted OR (95% CI) was 1.11 (1.02 to 1.21; p=0.017). For a SD slower choice reaction time at baseline, the multivariate-adjusted OR was 1.11 (1.00 to 1.24; p=0.048). In the sample with imputed data, ORs were very similar to those obtained in those with complete data.

**Table 2:** ORs (95% CIs) for psychological distress at follow-up for a SD increase in simple or choice reaction time at baseline in those with complete data (n=3088) and in those with imputed covariate data (n=3545)

	Simple reaction time, per SD	p Value	Choice reaction time, per SD	p Value
	OR (95% CI)		OR (95% CI)	
*Sample with complete data (n=3088)*				
Adjustments				
Unadjusted	1.14 (1.06 to 1.23)	<0.001	1.13 (1.04 to 1.22)	0.002
Age, sex, baseline GHQ score	1.12 (1.03 to 1.21)	0.007	1.14 (1.03 to 1.25)	0.011
Age, sex, baseline GHQ score, plus				
Social class and education	1.11 (1.02 to 1.21)	0.013	1.12 (1.01 to 1.24)	0.027
Health behaviours	1.11 (1.02 to 1.21)	0.012	1.13 (1.02 to 1.24)	0.018
Neuroticism	1.11 (1.02 to 1.21)	0.019	1.11 (1.01 to 1.22)	0.049
Chronic physical illnesses	1.12 (1.03 to 1.21)	0.009	1.13 (1.02 to 1.24)	0.015
All of the above	1.11 (1.02 to 1.21)	0.017	1.11 (1.00 to 1.24)	0.048
*Sample with imputed covariates (n=3545)*				
Adjustments				
Unadjusted	1.13 (1.06 to 1.21)	<0.001	1.13 (1.05 to 1.21)	0.001
Age, sex, baseline GHQ score	1.10 (1.02 to 1.19)	0.012	1.14 (1.04 to 1.24)	0.006
Age, sex, baseline GHQ score, plus				
Social class and education	1.09 (1.01 to 1.18)	0.025	1.12 (1.02 to 1.24)	0.015
Health behaviours	1.10 (1.02 to 1.19)	0.013	1.13 (1.03 to 1.24)	0.008
Neuroticism	1.09 (1.01 to 1.19)	0.027	1.11 (1.02 to 1.22)	0.027
Chronic physical illnesses	1.10 (1.02 to 1.19)	0.017	1.13 (1.03 to 1.24)	0.009
All of the above	1.10 (1.01 to 1.19)	0.024	1.11 (1.01 to 1.23)	0.031

GHQ, General Health Questionnaire.

We examined whether the association between simple or choice reaction time at baseline and psychological distress at follow-up varied by age (<35, 35–55, >55) or social class but there was no evidence of this (p for interaction terms >0.3).

People who had slower simple or choice reaction times also tended to have more variable reaction times (as indicated by the SDs of the reaction time measures): the correlation between either simple or choice mean reaction time and their respective mean SD was 0.63 (p<0.001). Greater intraindividual variability in simple or choice reaction was associated with a significantly increased risk of onset of psychological distress in analyses adjusted for age, sex and baseline GHQ score, but these associations did not persist after adjustment for mean reaction time (both p≥0.9).

We carried out an additional analysis looking at risk of distress in the subset of the sample who scored <5 on the GHQ at baseline; results were similar but weaker (see online supplementary material).

## Discussion

In this 7-year longitudinal study of a nationwide sample of UK adults, slower simple or choice reaction time at baseline was associated with an increased risk of psychological distress at follow-up as defined by scores of 5 or over on the GHQ-30. This association persisted, only slightly attenuated, after adjustment for a range of potential confounding factors, including baseline GHQ score, age, neuroticism, health behaviours, number of chronic physical illnesses, social class and educational attainment.

Only one previous study has examined the relationship between reaction time and later risk of psychological distress. This study, based on 705 16-year olds, found that slower choice reaction time was associated with higher scores on the 12-item GHQ and on the depression and anxiety subscales of the Hospital Anxiety and Depression Scale (HADS) at age 36 years; these associations were not explained by baseline levels of psychological distress or other potential confounding factors.^7^ One limitation of this latter study was that there was little variance in psychological distress among the sample at age 36: most of the respondents had low scores on the GHQ and the HADS. Effect sizes, as measured by difference in logged GHQ score and square-root transformed HADS anxiety and depression scores per SD slower choice reaction time, were small.^7^ In the current, larger study, where there was much more variation in GHQ scores, our findings provide further evidence that slower reaction time (whether assessed using either simple or choice reaction time) may be a risk factor for later onset of psychological distress. In contrast to the earlier study which was restricted to a single age cohort,^7^ our current sample was based on nearly the whole adult age range (18–99 years). We found no evidence that the relation between simple or choice reaction time and later psychological distress varied significantly by age.

Cross-sectional studies, mostly based on clinical samples, have found that people with more severe depressive symptoms tend to have slower reaction times and other cognitive deficits.^6^ ^21^ ^22^ These findings have generally been interpreted as indicating that the presence of depression can cause psychomotor slowing and have an adverse effect on task performance. Our observations here, coupled with those in the longitudinal study of adolescents,^7^ suggest that slower processing time may in fact be a risk factor for the onset of psychological distress. The fact that the association between reaction time and psychological distress at follow-up was little attenuated by adjustment for total GHQ score at baseline makes it very unlikely that the association is just a reflection of stable reaction time tracking stable low mood.

There is now considerable evidence that lower scores on tests of intelligence in youth tend to be associated with an increase in the risk of later diagnosis with depression or anxiety and of reporting symptoms of these disorders.^8–13^ ^23^ Reaction time is moderately highly correlated with intelligence^14^ and there is some evidence that it accounts to a large extent for the association between lower intelligence and increased risk of premature death.^24^ Reaction time tests are quicker to perform than tests of intelligence and have simple instructions. The extent to which less efficient processing of information explains the associations between lower intelligence and poorer mental health needs to be investigated in future studies.

The strengths of our study are its size, the fact that the sample was designed to be representative of the UK adult population and the availability of data on a range of potential confounding factors, especially mood state at baseline. One limitation is that recorded data on prescribed medication for each participant were restricted to any two drugs that potentially affect blood pressure or lung function, so we had no information on use of antidepressant drugs and data on sedative use are likely to be incomplete. We were therefore unable to take account of their potential influence on reaction time at baseline. A further potential weakness is that of those who were interviewed in the initial survey, only 40% completed the GHQ at follow-up 7 years later and only 34% had complete data on GHQ score at follow-up and all the baseline covariates. Based on results from multiple-imputation analyses, we found no evidence that our estimates based on those with complete data were biased by the exclusion of those with missing data on covariates, but we did not impute missing data on the outcome, so cannot rule out the possibility that loss to follow-up may mean that the associations we found underestimate the true association between reaction time and risk of psychological distress.

In this prospective study of men and women aged 18 and over from a nationwide UK sample, we found that slower processing speed, as indexed by longer simple or choice reaction time, was associated with an increased risk of psychological distress 7 years later. Further prospective studies of the relation between reaction time and mental health outcomes are needed to assess whether slower processing speed is indeed a risk factor for onset of mental disorders and the extent to which it explains previously demonstrated associations between lower intelligence and poorer mental health.

**What is already known on this subject**

- Evidence from cross-sectional studies suggests that processing speed tends to be slower in people who are depressed but the direction of effect is unclear.
- A recent study found that adolescents with slower processing speed as measured by reaction time had more symptoms of depression and anxiety 20 years later.

**What this study adds**

- In people aged 18 or over, slower processing speed, as indexed by longer simple or choice reaction time, was associated with an increased risk of psychological distress 7 years later.
- These associations persisted after adjustment for age, sex, health behaviours, education, social class, chronic illness, neuroticism and level of psychological distress at baseline.
- Slower processing speed may be a risk factor for the development of psychological distress.

## Supplementary Material

Web supplement
