# Supplementary material for: Reaction time and onset of psychological distress: the UK Health and Lifestyle Survey
Source: J Epidemiol Community Health. 2016 Feb 4;70(8):813–7. doi: 10.1136/jech-2015-206479 (PMC4975803; doi:10.1136/jech-2015-206479)
Supplement: Web supplement [file jech-2015-206479-s1.pdf]

Supplementary table: Rank order correlations between mean simple and choice reaction time[1] and the covariates at baseline and GHQ-30 score at follow-up

**Characteristics**	**Simple reaction time**	**Choice reaction time**
Age	0.24***	0.52***
Social class	0.19***	0.18***
Educational qualifications	-0.26***	-0.37***
Neuroticism	0.08***	0.05***
Ever smoked	0.01	0.06**
Units of alcohol	-0.11***	-0.14***
Fruit/vegetables consumption frequency	-0.09***	-0.04*
Hours of physical activity	-0.13***	-0.24***
Number of chronic physical illnesses	0.10***	0.19***
GHQ-30 score	0.07***	0.07***
GHQ-30 score at follow-up	0.07***	0.09***

***p<0.001, **p<0.01

[1]Larger values for reaction time mean slower (longer) reaction time

Supplementary material: We ran an additional analysis in which we excluded all those who scored ≥5 on the GHQ at baseline (30% of our original sample). In this subset of 2219 people, the variance in (and level of) the GHQ score at follow-up was markedly smaller than in our sample as a whole (mean (SD) 0.92 (1.25) *vs* 3.90 (5.87)), and as might be expected given this, age- and sex-adjusted odds ratios for incident psychological distress per SD increment in simple or choice reaction time were weaker than those obtained in our sample as a whole: 1.11 (1.00, 1.24) and 1.11 (0.99, 1.28) respectively *vs* 1.16 (1.08, 1.26) and 1.21 (1.11, 1.33).
